# Androgens as the “old age stick” in skeletal muscle

**DOI:** 10.1186/s12964-025-02163-6

**Published:** 2025-04-03

**Authors:** Giulia Gentile, Ferdinando De Stefano, Carmela Sorrentino, Rosa D’Angiolo, Carmine Lauretta, Pia Giovannelli, Antimo Migliaccio, Gabriella Castoria, Marzia Di Donato

**Affiliations:** Department of Precision Medicine, University of Campania “Luigi Vanvitelli”, Via L. De Crecchio 7, Naples, 80138 Italy

**Keywords:** Androgens, Androgen receptor, Aging, Sarcopenia

## Abstract

Aging is associated with a reduction in skeletal muscle fiber size and number, leading to a decline in physical function and structural integrity—a condition known as sarcopenia. This syndrome is further characterized by elevated levels of inflammatory mediators that promote skeletal muscle catabolism and reduce anabolic signaling.

Androgens are involved in various biological processes, including the maintenance, homeostasis and trophism of skeletal muscle mass. The decline in androgen levels contributes, indeed, to androgen deficiency in aging people. Such clinical syndrome exacerbates the muscle loss and fosters sarcopenia progression. Nevertheless, the mechanism(s) by which the reduction in androgen levels influences sarcopenia risk and progression remains debated and the therapeutic benefits of androgen-based interventions are still unclear. Given the significant societal and economic impacts of sarcopenia, investigating the androgen/androgen receptor axis in skeletal muscle function is essential to enhance treatment efficacy and reduce healthcare costs.

This review summarizes current knowledge on the role of male hormones and their-dependent signaling pathways in sarcopenia. We also highlight the cellular and molecular features of this condition and discuss the mechanisms by which androgens preserve the muscle homeostasis. The *pros* and *cons* of clinical strategies and emerging therapies aimed at mitigating muscle degeneration and aging-related decline are also presented.

## Introduction

Skeletal muscle constitutes approximately 45% and 35% of lean body mass in men and women, respectively. It comprises elongated, multinucleated cells called myofibers, which are specialized for contraction [1]. Each myofiber contains thousands of myofibrils made up of billions of myofilaments, predominantly actin and myosin. These myofilaments organize into sarcomeres, the fundamental contractile units of skeletal muscle.

Beyond its role in locomotion, skeletal muscle is critical for basal metabolism, as it stores amino acids, carbohydrates, fatty acids and other substrates. Moreover, it plays a central role in glucose homeostasis by regulating insulin-stimulated glucose uptake and postprandial glucose disposal in humans and rodents [2, 3].

Since the fourth decade of life, skeletal muscle undergoes a progressive decline in cross-sectional area, attributed to reductions in both fiber size and number [4–6]. Such condition, also termed sarcopenia [7], was firstly identified in the late 1980s as a significant clinical issue in aging populations. Only recently, however, it has been recognized as a disease by the International Classification of Diseases (ICD-10; [8]). Sarcopenia affects approximately 5% of individuals aged 65 and up to 50% of those over 80 [9]. It leads to reduced mobility, physical disability, slow gait, loss of independence, and poor quality of life [10]. Despite its incidence, sarcopenia often remains neglected and undertreated, contributing to increased healthcare costs and economic strain. Notably, the sedentary lifestyle associated with sarcopenia exacerbates metabolic disorders, such as insulin resistance and hepatic steatosis, further diminishing both the expectancy and quality of life [11].

The growing aging population has intensified interest among researchers, clinicians and pharmaceutical companies in addressing the need of effective treatments for sarcopenia. The condition is multifactorial, involving systemic inflammation, altered growth factor signaling, and reduced hormone levels, particularly androgens, estrogens and progestins. By modulating metabolism, maintenance, strength, growth, hypertrophy and repair [9], these hormones support skeletal muscle health. Their effects are mediated through the androgen receptor (AR), estrogen receptors (ER)-α or β and progesterone receptor (PR), respectively, which act on various muscle components, including satellite cells, myoblasts, and myocytes [12–16]. The decline in skeletal muscle mass is closely linked to andropause and menopause, which are marked by reduced sex steroid and growth hormone levels [17, 18].

In this review, we will focus on the role of androgens and their cognate receptors in skeletal muscle homeostasis and trophism. The interplay between androgen/AR signaling and other sex steroid- or growth factor-dependent pathways will be also presented, together with its putative contribution to age-related frailty. The benefits and limits of anabolic therapies based on testosterone replacement or use of new selective androgen receptor modulators (SARMs) will be lastly discussed.

### Molecular mechanism of the androgen/androgen receptor (AR) axis in skeletal muscle

Sex steroids control the development of primary and secondary sex characteristics as well as the physiology of reproductive system [19]. They are synthesized by gonads and adrenal glands or derive from the conversion of other sex steroids in some tissues, including liver and fat [20]. These hormones share the same chemical backbone, derived from a common biosynthesis pathway stemming from cholesterol. According to the number of carbon atoms, they are classified into androgens (19 C), estrogens (18 C) and progestins (21 C) [21].

Androgens establish and maintain the male phenotype, leading to differentiation and growth of the male reproductive organs, regulation of spermatogenesis as well as the control of male sexual behavior [22]. They also exert important functions in several non-reproductive tissues, including muscle, adipose, bone and brain [23]. In skeletal muscle, androgens enhance protein synthesis and intracellular amino acid recycling, promote myofiber hypertrophy, increase glycolytic flux and the number of myonuclei, and induce the proliferation of satellite cells [24–27]. The latter represent the predominant sites of androgenic action due to their high AR expression [13]. In satellite cells, androgens upregulate the expression of their own receptor, stabilize the existing pool of AR or enhance its de novo synthesis, thus increasing the sensitivity of satellite cells to their action [28]. Androgens also induce changes in body composition through an increase in lean body mass and a decrease in fat mass, likely because of their effect on differentiation of pluripotent precursor cells into the myogenic lineage [23]. In myofibers, androgens enhance muscle mass and strength as well as glucose uptake, while limiting the amino acid catabolism and the uptake of free fatty acids [29]. These effects likely account for muscle-related diseases upon a deficiency or decline in the androgen levels.

The androgen action is mediated by the classic AR, a member of the steroid receptor superfamily. The receptor is a 110 KDa protein structurally composed of an N-terminal domain, a DNA-binding domain and a ligand-binding domain. The latter is commonly activated by testosterone and its powerful metabolite 5α-dihydrotestosterone (DHT) [30]. A range of hormones, growth factors and peptides might, however, indirectly activate or inactivate AR [23]. Once coupled to the ligand, AR regulates in a transcriptional or non-transcriptional way quite different cellular responses, such as gene transcription, epigenetic modifications and extranuclear signaling pathways. Integration of these machineries ultimately controls cell cycle, survival, migration, differentiation and metabolic changes in a plaethora of cell types [23, 31–34].

### Transcriptional actions mediated by AR in skeletal muscle cells

AR regulates the expression of androgen-responsive genes by binding specific DNA sequences, the androgen-responsive elements (ARE). They are located near or within the promoters or enhancers of the target genes, and a significant number of ARE has been identified in different target tissues [35–37].

The impact of the androgen–AR signaling transcriptional pathway on muscle function and sarcopenia has been outlined by several papers. DNA binding studies by ChIP-on-ChIP and ChIp-qPCR analyses in cell cultures have identified novel AR targets, including genes and micro-RNAs implicated in muscle differentiation and function. Among them, binding sequences for the myocyte enhancer factor 2 (Mef2) family of transcription factors were found enriched in the AR-bound regions and several Mef2c (myocyte enhancer factor 2 C)-dependent genes were identified as direct targets of AR. These findings suggested a functional interaction between Mef2c and AR in skeletal muscle development [38].

Muscle-specific AR knockout (ARKO) mice models using the muscle creatine kinase (MCK)-Cre [39] or the Human α-skeletal actin (HSA)-Cre promoters [40] were developed to study the AR transcriptional function in muscle tissue, yielding a wide range of results from maintenance of muscle mass and fiber type regulation [39] to the strength of limb muscle [40]. ARKO mice with both the promoters showed minimal changes in skeletal muscle mass. MCK-Cre knockout mice did not exhibit alteration in muscle strength or fatigue, while HSA-Cre knockout mice showed reduced muscle strength. In MCK-Cre knockout mice, the soleus muscle showed a transformation from fast to slow of fiber types upon AR knockdown, suggesting that myocytic AR plays a role in regulating the muscle fiber types. Similarly, myoblast determination protein 1 (MyoD)-iCre-mediated satellite cell-specific ARKO mice exhibited both fiber conversion and decreased grip strength [41]. Again, the AR requirement for androgen-induced muscle strength has been elucidated in myofiber-specific ARKO (cARKO) mouse model. Specific supply of DHT in myofiber enhanced grip strength in control but not in cARKO mouse. Subsequent transcriptomic analyses identified a fast-twitch muscle-specific *Myosin light-chain kinase 4 (*Mylk4) variant as an AR target gene. Mylk4 knockout mice exhibited, indeed, reduced isometric torque and passive muscle stiffness, attributed to decreased phosphorylation of myomesin 1, a structural protein integral to sarcomere integrity. These findings delineate a novel AR-dependent axis crucial for muscle strength, offering potential therapeutic targets for muscle-wasting conditions [42].

By using a fast-twitch muscle-specific ARKO [fmARKO] mouse model, it has been consistently reported that AR deletion does not affect the muscle mass, while reducing the muscle strength and fatigue resistance in young male mice. Muscle overall function declined in middle-age male fmARKO mice, thus mimicking the scenario of sarcopenia progression. Interestingly, in this animal model downregulation of polyamine biosynthesis-related genes was revealed by microarray and gene ontology approaches, indicating a role for AR transcriptional pathway in maintaining muscle composition and function [43]. These findings are consistent with a previous report showing that skeletal muscle from older mice exhibit a reduction in levels of polyamines, whose biosynthesis is regulated by the AR transcriptional activity [44]. These reports have provided new hints into the role of AR in pathophysiology of skeletal muscle, as the loss of polyamines is involved in muscle wasting and sarcopenia [44, 45]. They have also suggested new approaches (i.e., polyamine’s supply) in the treatment of sarcopenia [43].

Mice model carrying a selective ablation of AR in myofibers (AR^skm−/y^ mice) exhibits a decrease of glucose uptake and glycolytic activity, thus contributing to early-onset type 2 diabetes. Combined cistromic and transcriptomic analyses have shown that AR expressed in myofibers controls the expression of transcription factors and genes involved in glycolysis, fatty acid metabolism, polyamine biosynthesis, reactive oxygen species (ROS) scavenging and cytoskeleton remodeling of skeletal muscle. Genes associated with glucose metabolism, including fructose-bisphosphatase 2 (Fbp2), pyruvate carboxylase (Pcx), and 6-phosphofructo-2-kinase/fructose-2,6-biphosphatase 3 (Pfkfb3), have been identified as direct AR targets. Additionally, impairment of fatty acid metabolism and oxidative phosphorylation has been detected in the muscles of AR^skm−/y^ mice. The finding that AR directly activates the transcription of genes involved in glycolysis, oxidative metabolism and muscle contraction, further underscores the role of the receptor in skeletal muscle pathophysiology [37].

Other factors, however, might influence the AR-dependent transcriptional events in skeletal muscle. Co-regulators, which include co-activators and co-repressors, affect a wide range of responses mediated by AR in target tissues (*reviewed in* [46, 47]). Their aberrant activity, likely caused by mutation or altered expression levels, might contribute to pathogenesis of AR-related diseases.

Given the importance of cytoskeleton components and its dynamic changes in architecture, mechanical and signaling functions of skeleton muscle [48], it is essential to pay attention on some actin-binding proteins, which are considered as putative AR co-activators in skeletal muscle cells. Gelsolin (GSN), also called Actin-depolymerizing factor (ADF) or brevin, for instance, interacts with AR in an androgen-dependent manner and enhances AR transactivation by co-localizing with the receptor when it enters the nuclei. As such, gelsolin would contribute to myotube’s maturation [49]. Again, the supervillin, a 205 kDa actin-binding protein, structurally homologue to gelsolin and villin, together with its muscle specific isoform, archvillin, interact and transactivate AR [50, 51]. Of note, archvillin increases during myogenesis and contributes to myotubes formation [51]. The hydrogen peroxide-inducible clone-5 (Hic-5), AR co-activator Hic-5 (ARA55) and paxillin (PXN) share similarities and serve as AR coactivators [52–54]. The focal adhesion scaffold protein, PXN shuttles between focal adhesion sites and the nuclear matrix, interacts with AR and glucocorticoid receptor (GR) through the C-terminal LIM domain and functions as coactivator of both the receptors in prostate cancer [53]. Despite PXN represents a valuable link between nuclear and extranuclear androgen action in prostate cancer cells [55], no similar evidence has been so far reported in skeletal muscle cells.

Other AR co-activators have been identified, which facilitate chromatin remodeling, recruit transcriptional machinery or stabilize AR-DNA interactions. Among them, the p160/steroid receptor coactivator (SRC) family comprises three pleiotropic coregulators (SRC-1, SRC-2 (TIF2/GRIP1), and SRC-3 (AIB1); otherwise known as nuclear receptor coactivators (NCOA1, NCOA2, and NCOA3, respectively). They recruit histone acetyltransferases to promote AR transcriptional activation and enhance anabolic signaling as well as hypertrophic responses to androgens. Specifically, SRC-1 promotes energy expenditure, SRC-2 supports energy storage and mitochondrial function, while SRC-3 regulates energy substrate utilization and affects muscle endurance [56, 57]. Again, the peroxisome proliferator-activated receptor gamma coactivator-1 (PGC-1) family, comprising PGC-1α, PGC-1β, and PGC-1-related coactivator (PRC), plays a pivotal role in regulating mitochondrial biogenesis, oxidative metabolism and energy homeostasis across various tissues, including skeletal muscle [58]. PGC-1α is a master regulator of mitochondrial biogenesis and interacts with AR and other steroid receptors to modulate gene expression in skeletal muscle. By enhancing AR transcriptional activity, PGC-1α, for instance, influences muscle growth and function [59]. On the other hand, the functions of AR might be negatively regulated by co-repressors that often recruit histone deacetylases (HDACs) to compact chromatin. In such a way, they inhibit gene transcription. Among them, the Nuclear Receptor Co-Repressor (NCoR) stabilizes the closed chromatin state [60] and regulates catabolic processes as well as AR signaling during stress or inflammation of skeletal muscle [61].

However, the ligand availability and the AR protein levels regulating the AR-dependent transcriptional activity cannot be neglected. The decline in muscle functions correlates, indeed, with the gradual decrease in circulating androgen levels in elderly males *(reviewed* in [62]). It occurs in parallel with declines in skeletal muscle mass [63, 64], strength [65, 66] and physical performance [67]. Therefore, the reduction in androgen levels might account for a decline in AR-transcriptional activity. Previous studies have consistently shown that low androgen levels impair the AR-mediated gene transcription in quite different cell types [68, 69].

Concerning the AR content in skeletal muscle cells, very conflicting results have been previously obtained by AR mRNA analysis in young and adult males, under basal conditions or after exercise [70–74]. As stated above [37], a reduction in muscle AR levels has been detected during age-related muscle loss [75] and a decrease in AR protein levels has been reported in skeletal muscle biopsies from older compared to young people [76]. Of note, AR nuclear translocation and ARE-dependent transcription can be detected in various non-reproductive cells, provided the expression of significant AR amounts [69, 77]. In this regards, it should be also mentioned that AR degradation by ubiquitin-proteasome pathway impairs the receptor transcriptional activity [78, 79] and a significant increase in ubiquitin ligase expression promotes muscle wasting and impairs muscle contractility in older people [80–84]. Exploitation of the link between AR degradation, ubiquitin ligase system and receptor-dependent gene transcription deserves, hence, further in depth investigation in skeletal muscle cells.

### Non-transcriptional actions mediated by AR in skeletal muscle cells

In addition to controlling gene expression, androgens trigger rapid responses in the extranuclear compartment of target tissues. Activation of a cascade of intracellular effectors, including protein kinase A (PKA), protein kinase C (PKC), phospholipase C (PLC), tyrosine sarcoma kinase (Src), phosphoinositide 3-kinases (PI3Ks) or mitogen-activated protein kinases (MAPKs) then follows. These events ultimately control a range of responses, including cell cycle progression and survival, cytoskeleton changes and motility as well as transcriptional and translational activities (reviewed in [85, 86]).

Many years ago, it was reported that androgens trigger very rapid responses involving membrane-linked signal transduction pathways in skeletal muscle cells, leading to an early increase of calcium, with a concomitant increase in inositol triphosphate (IP3) concentration [87]. Additional studies showed that the rapid androgen action involves a pertussis toxin (PTX) sensitive-G (Gαi/Gαo) protein-linked receptor at the plasma membrane and leads to IP3-mediated calcium signaling the Ras/mitogen-activated extracellular signal-regulated kinase (MEK)/ extracellular signal-regulated kinase (ERK) pathway activation as well in muscle cells [88]. Androgen stimulation of L6 myoblasts, lacking the classical AR and stably expressing human wild type AR (L6.AR cells), triggers activation of both mammalian target of rapamycin (mTOR) and ERK1/2 [89]. Subsequent studies have shown that androgens foster proliferation and differentiation of L6 myoblasts through a G-protein–coupled receptor [90]. Additional findings on rapid, non-nuclear events activated by androgens in skeletal muscle cells have shown that testosterone loss suppresses myofibrillar protein synthesis through Akt/mTOR signaling and activates muscle Forkhead box protein O3 (FoxO3a) and its transcriptional targets in mouse skeletal muscle. Androgen supply restores Akt/mTORC1/FoxO3a signaling activation in castrated mice [91]. These findings raise the question about the mechanism of PI3K signaling activation by androgens. It might occur through a direct interaction between AR and the p85α PI3K regulatory subunit [92, 93]. However, other mechanisms, including the involvement of focal adhesion kinase (FAK), cannot be ruled out, given the rapid FAK activation by androgens in skeletal muscle cells [76]. The involvement of insulin receptor substrates (IRS), mainly IRS-1 and IRS-2, in the androgen activation of PI3K signalling cannot be excluded. By stimulating the expression or activity of IRSs through transcriptional regulation or other androgen-activated pathways, AR might mediate PI3K signalling activation [94, 95]. Of note, the androgen modulation of AMP-activated protein kinase (AMPK) activity might also indirectly influence PI3K signaling, thus promoting glucose uptake, protein synthesis, and mitochondrial biogenesis in skeletal muscle [96, 97]. Key metabolic pathways, overlapping with PI3K/Akt signaling, modulates AMPK. By increasing the expression of IRS-1 and IRS-2, AMPK might amplify the effects of insulin mediated by PI3K/Akt signaling activation. The activation of PGC-1α by AMPK upregulates the genes involved in mitochondrial function and energy metabolism, while indirectly enhancing PI3K/Akt signaling [98]. The link between AMPK and PI3K pathways seems very important in muscle cells physiology, as androgens might promote muscle growth via PI3K/Akt, and energy metabolism through AMPK [99].

The intersection between AR and growth factors has been also reported in skeletal muscle cells. Insulin-like growth factor 1 (IGF-1), a muscle growth-promoting factor, is mainly produced in liver upon growth factor stimulation [100], or by skeletal muscle cells on androgen stimulation [101]. AR expressed in mesenchymal progenitors maintains skeletal muscle mass via regulation of IGF-1 [102], further pointing to the connection between IGF-1 and AR. IGF-1 activates the PI3K-Akt pathway through the binding with its receptor, IGF-1R (insulin-like growth factor 1 receptor), and is an important mediator of androgen action in skeletal muscle [103]. Androgen depletion by castration decreases muscle IGF-1 mRNA expression in mice, accounting for muscle wasting associated with a decline in androgen levels (i.e., hypogonadism or aging). Androgen supply reverses this effect [91] and enhances PI3K activation by IGF-1. Consequently, Akt phosphorylation and protein synthesis follow through activation of mTOR, phosphorylation of the translation initiation factor 4E-binding protein 1 (4E-BP1) and the simultaneous release of the eukaryotic translation initiation factor 4E (eIF4E), as well as the activation of ribosomal protein S6 Kinase beta 1 (S6K1). On the other, Akt activation triggers the phosphorylation of FoxO, promoting the export and sequestration of phosphorylated FoxO proteins in the cytosol. In this manner, AKT signaling suppresses the expression of FoxO target genes involved in apoptosis, cell-cycle arrest, catabolism and growth inhibition, while stimulating glucose uptake, glycogen synthesis and protein synthesis.

Additionally, FoxO transcription factors promote the expression of atrogin-1/muscle atrophy F-box (MAFbx) and muscle RING finger 1 (MuRF1), two muscle-specific E3 ubiquitin ligase enzymes. As above discussed in this manuscript, the upregulation of these enzymes facilitates the ubiquitination of target proteins and their degradation via the proteasome, contributing to muscle atrophy. In summary, the FoxO family regulates muscle cell growth, differentiation, autophagy, apoptosis and energy metabolism in skeletal muscle. By mediating different processes, the FoxO family significantly impacts skeletal muscle homeostasis and age-related diseases [104, 105].

The arguments put forward here raise the question of the identity and localization of the AR mediating these responses. Among the various AR variants so far identified in target human tissues, a naturally occurring variant of the AR, also named AR45, has been detected in heart and skeletal muscle. It lacks the entire region codified by exon 1 of the AR gene and is made up of the AR DNA-binding domain, hinge region and ligand-binding domain, preceded by a novel seven amino-acid long N-terminal extension. This variant might modulate the functions of wild type AR, thus making more complex the mode of androgen action in human skeletal muscle [106]. Androgens might also bind a classical receptor poised near the cell membrane or in cytoplasm of skeletal muscle cells [76, 107]. Additionally, a membrane AR (mAR), distinct from the classical receptor and associated with the plasma membrane or localized in caveolae, has been detected in C2C12 skeletal muscle cells [108]. Despite the discovery of the mAR in various cell types, including macrophages and T lymphocytes [109, 110], prostate [111] as well as breast [112] cancer cells, the identity of such receptor remains still debated. While we were reviewing our manuscript, new interesting findings have identified the adhesion G-protein coupled receptor (aGPCR) GPR133 (also known as ADGRD1) as an androgen membrane receptor for DHT that modulates muscle strength [113].

We have reported that classical, cytoplasmic AR forms a bipartite complex with Filamin (Fln) A in muscle biopsies from young subjects but not from the older adults, suggesting a putative role for this complex in muscle trophism. Experiments in C2C12 cells have shown that androgens prevent the senescence induced by oxidative stress through the assembly of such complex. This event leads to activation of various downstream signaling effectors, including FAK, PXN, Rac 1 and ERK [76]. The finding that the androgen induced AR/FlnA complex prevents the senescence in C2C12 myoblasts [76], points to the functional role of FlnA in age-related diseases and senescence. It has been consistently shown that genetic variations and FlnA abundance induce tau aggregation and tauopathies [114]. Inhibition of FlnA interaction with dynamin-related protein 1 (Drp1), a modulator of mitochondrial dynamics, attenuates cardiomyocyte senescence after myocardial infarction [115]. Again, the loss of FlnA, in combination with formin 2 knock-out, leads to smaller body size, thinned muscle and skeletal wall as well as defects in cardiac valve in a mouse model [116]. Noticeably, the signaling effectors rapidly activated by androgens in C2C12 cells are involved in muscle pathophysiology, as Rac 1 controls the late muscle development [117], while FAK is involved in skeletal muscle cell development and homeostasis [118]. At last, a role for MAPK activation in muscle protein synthesis and cachectic or sarcopenic conditions has been reported [119].

The intersection between androgen and myostatin pathway deserves consideration. Myostatin, a member of the transforming growth factor-β (TGF-β) superfamily, is specifically expressed in skeletal muscle, where it binds its cognate receptor (activin receptors type I and type II). On ligand stimulation, the tetramerization of the receptor complex allows the phosphorylation of small mothers against decapentaplegic (SMAD) proteins in cytoplasm. Once activated, the SMAD family member 4 (SMAD4) enters the nuclei and regulates the expression of genes inhibiting myoblast proliferation and differentiation, such as paired Box 3 (Pax3) and MyoD [120]. Androgens inhibit the myostatin axis in skeletal muscle cells through follistatin, an antagonist of several members of the TGF-β superfamily, including myostatin. Follistatin blocks, indeed, the binding of myostatin to its receptor, thus preventing its activity. In such a way, androgens might re-establish the correct skeletal muscle homeostasis [121].

The androgen-positive effects on muscle growth might be also elicited through the Notch signaling. Androgens, indeed, activate Notch signaling through the MAPK cascade activation [122]. By activating Notch signaling in aged mice, testosterone inhibits the age-associated oxidative stress, muscle cell apoptosis and myostatin expression [123].

From the findings so far presented, it appears evident that further experimental data and models are required for a better understanding of the intricate network activated and/or modulated by androgens in skeletal muscle cells.

**Fig. 1:**
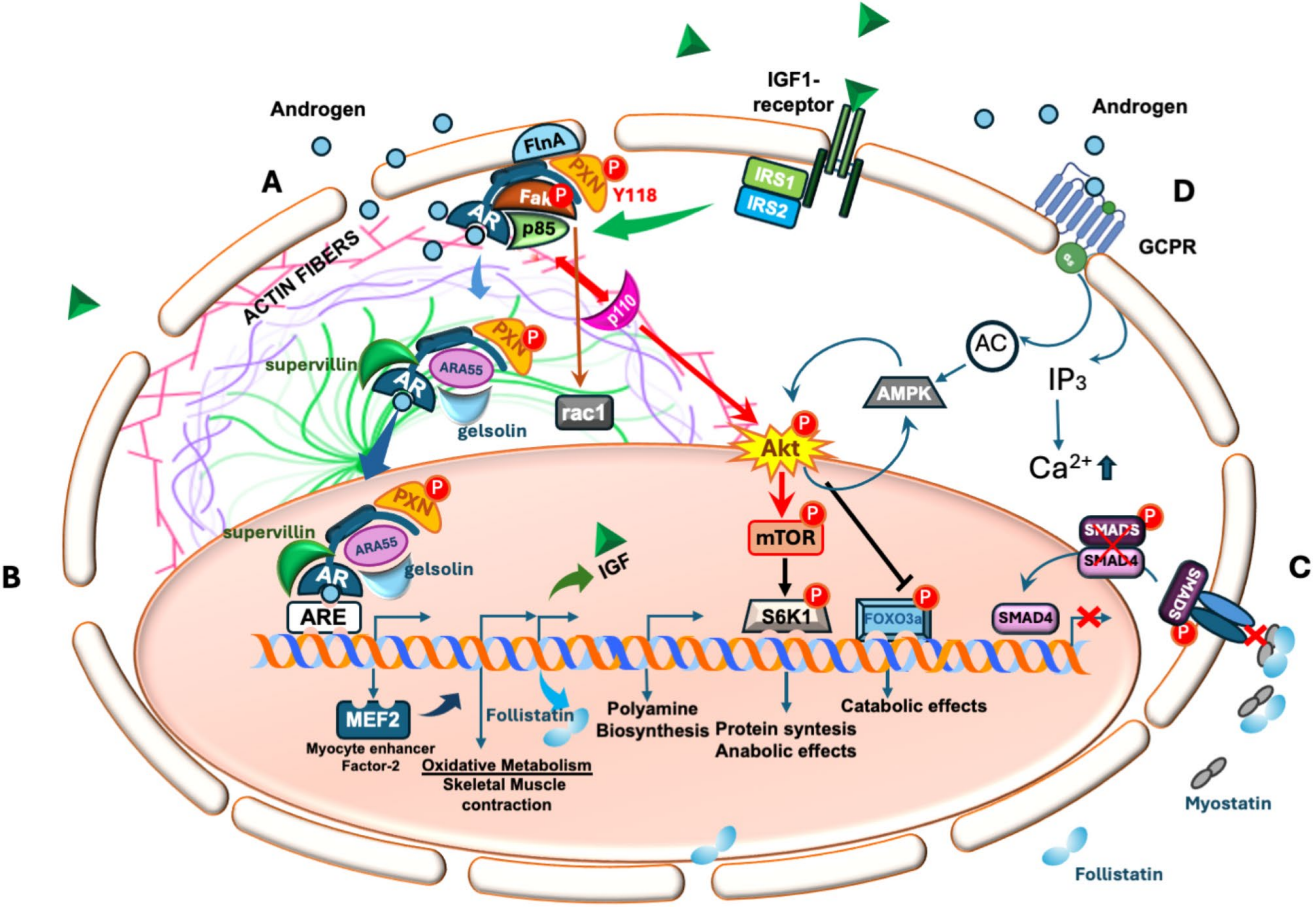
Non-transcriptional and transcriptional events activated by AR in skeletal muscle cells. It illustrates the key partners of the androgen receptor (AR) identified in skeletal muscle cells and mediating transcriptional or non-transcriptional androgen actions. (**A**) Upon crossing the plasma membrane, androgens bind to AR in cytoplasm of skeletal muscle cells. This event leads to the assembly of a binary complex made up of AR and Filamin A (FlnA), triggering the activation of downstream signaling pathways, including Focal Adhesion Kinase (FAK), paxillin, and Rac1. Additionally, androgen stimulation allows AR to interact with p85, the regulatory subunit of PI3K, leading to its activation. PI3K activation may also occur *via* a FAK-mediated mechanism. The downstream mediators of IGF-1 signaling, insulin receptor substrates 1 and 2 (IRS-1 and IRS-2) further contribute to PI3K activation. Whatever the upstream mechanism, activated PI3K, in turn, phosphorylates and activates Akt. This event results in increased protein synthesis and anabolic effects via mTOR and S6K1 phosphorylation, on one hand. On the other, Akt suppresses catabolic effects by phosphorylating FoxO3a, thereby inhibiting its transcriptional activity. (**B**) Upon ligand binding, AR enters the nuclei, where it interacts with co-activators such as paxillin, gelsolin, supervillin, and ARA55. AR then binds to androgen-responsive elements (AREs) to regulate the transcription of genes involved in muscle development (MEF2) as well as glycolysis, oxidative metabolism, muscle contraction and polyamine biosynthesis. (**C**) Myostatin binds to its receptor, thus inducing receptor tetramerization and the subsequent phosphorylation of cytoplasmic SMAD proteins. Once phosphorylated, SMAD4 translocates to the nucleus, where it inhibits the transcription of genes essential for myoblast proliferation and differentiation. (**B**,** C**). By activating IGF-1 axis (as depicted in A), androgens, positively influence the expression of follistatin. By preventing the binding of myostatin to the receptor and, consequently, its activation, follistatin counteracts the activity of myostatin, thus promoting oxidative metabolism and skeletal muscle contraction. (**D**) Androgen binding to GPCRs at the plasma membrane triggers adenylate cyclase (AC), leading to increased intracellular levels of inositol triphosphate (IP3) and calcium (Ca²⁺). This pathway also promotes AMP-activated protein kinase (AMPK) activation, which modulates the PI3K/Akt circuit. Akt might also modulate AMPK activity, further integrating the androgen signaling activation in skeletal muscle cells

Figure 1 aims to depict this scenario.

### Androgens, aging and sarcopenia: interconnected mechanisms and clinical implications

As above mentioned, sarcopenia is characterized by a slow and progressive decrease in muscle mass and function, leading to reduced mobility, physical disability, increased risk of falls and fractures and consequently to a loss of independence. As such, the syndrome is linked to a poor quality of life in aging people and is a major burden for the national sanitary systems [124].

Sarcopenia is a multifactorial disease mainly caused by endocrine system impairment [125], low grade inflammation, oxidative stress, motoneuron deficiency, redistribution of body fat and others disorders [126, 127]. As androgens are essential for maintenance of muscle mass and strength [28, 128] and exogenous testosterone supply increases the muscle mass and leg strength in eugonadal young and aging men [129, 130], the role of androgen/AR signaling in sarcopenia pathogenesis appears evident. Clinical studies have indicated a slight, but significant, disparity in the incidence of sarcopenia in men as compared with women (26.8% vs. 22.6% respectively). Such disparity widens in people over 80 years old (53% and 31% in men and in women, respectively) [64]. A 50% decrease in free-testosterone levels is associated with a 1.4-fold higher probability of frailty and a 1.55-fold higher risk of sarcopenia in men [131]. However, while the association between low testosterone levels and frailty is well established in men [132], evidence in women is more limited, although a similar correlation has been proposed [133]. Nonetheless, women experience more pronounced bone and muscle decay [134], with symptoms such as low back pain, joint or muscle pain, cognitive impairment, fatigue and sleep disturbances [135]. Sex-dependent differences in skeletal muscle metabolism have been, however, highlighted by recent transcriptomic and functional studies. These differences rely on glycolysis in male, while shifting towards mitochondrial fatty acid β-oxidation in female [136]. Expression of phosphofructokinase-2 would enhance glycolysis in type IIB fast-twitch fibers in males, while estradiol-induced pyruvate dehydrogenase kinase 4 would shift muscle female metabolism towards fatty acid oxidation [137].

Of note, the aerobic capacity is also reduced in elderly people and such event might foster sarcopenia progression [138]. The finding that testosterone treatment ameliorates the muscle aerobic capacity further supports the role of male hormones in this disorder [139]. Clinical trials have consistently reported a significant improvement of the 6-minutes walking distance test, stair climb power, leg press strength and power on androgen treatment, once again indicating a dangerous liaison between the androgen decline and the loss of skeletal muscle trophism [127, 140].

Sarcopenia can be clinically classified in primary and secondary. The first one, predominantly related to aging, is characterized by hormonal deficiency and associated with apoptosis as well as mitochondrial dysfunction [141]. Secondary sarcopenia is, instead, related to different factors, such as skeletal muscle disuse, chronic diseases (e.g. heart or respiratory failure) or malnutrition [142]. Relevant to this paper, an increase in sarcopenia incidence together with an excess of adiposity [143] have been observed in prostate cancer patients treated with androgen deprivation therapy (ADT; [144]. A different form of secondary sarcopenia is, instead, associated with cachectic condition, which often occurs in cancer patients because of an unbalance between catabolic and anabolic processes. Unlike the ADT-induced sarcopenia, characterized by the loss of muscle tissue and fat gain, cachectic sarcopenia is accompanied by a loss of reserves in both muscle and adipose tissues [143, 145]. Noticeably, tumor cells produce high levels of interleukin-6 (IL-6), which lower the synthesis of muscle proteins and enhance muscle wasting [146]. The anti-IL-6 antibody, siltuximab, in combination with androgens or other anabolic steroids, slows down the cachexia in patients with hormone-insensitive cancers, which often exhibit a dramatic decrease in androgen levels [147]. Such therapeutic approach might not be useful, of course, in androgen-sensitive cancers.

Sarcopenia, however, rarely occurs alone, as it is often accompanied by other syndromes, such as osteoporosis, insulin-resistance, obesity and metabolic syndrome [148, 149]. Declining testosterone levels might even induce hypogonadism [150], thus creating a feedback-loop that further exacerbates these conditions. Androgens regulate bone resorption and matrix deposition, positively correlating with bone mineral density. Consequently, declining androgen levels can lead to osteoporosis, characterized by reduced bone mass, microarchitectural deterioration, and increased fracture risk [151, 152]. Again, obesity and insulin resistance has been reported in humans with low androgen/AR signaling activity, including hypogonadal men, ADT-treated prostate cancer and Kennedy’s disease patients. Moreover, patients with complete androgen-insensitivity syndrome, have higher propensity to develop obesity and abnormal insulin sensitivity than normal male subjects [153]. These findings support the role of androgen/AR axis in modulating the insulin responsiveness in males. By controlling fatty acid storage through lipoprotein lipase (LPL) activity, androgens also modulate adipose tissue function [154]. Patients with low testosterone levels show a higher storage of fatty acid in adipose tissue [155]. In turn, the leptin—produced by adipose tissue—inhibits androgen production, compromising the reproductive function in obese individuals. The adipokine secretion increase might significantly contribute to insulin resistance and development of type II diabetes, creating a vicious cycle in which dysfunction of adipose tissue induces hypogonadism, exacerbating testosterone sequestration and inactivation [156–158]. Further, the aging-related neuromuscular deterioration complicates the clinical picture [159], as testosterone maintains motor unit functionality and its decline is linked to reduced compound muscle action potentials, reflecting muscle atrophy [160]. At last, we cannot neglect the role of androgen/AR in inflammatory and immune responses. By reducing pro-inflammatory cytokines, such as tumor necrosis factor-α (TNF-α), IL-6, and interleukin-1β (IL-1β), male hormones exhibit anti-inflammatory properties, while simultaneously enhancing anti-inflammatory cytokines, such as interleukin-10 (IL-10; [161]). The role of IL-6 in cachexia-related sarcopenia has been previously discussed in this section and elevated levels of TNF-α, IL-6 as well C-reactive protein (CRP) have been detected in sarcopenic people, further suggesting a link between systemic inflammation and muscle degeneration [162, 163]. Moderate exercise counteracts, indeed, inflammation, insulin resistance and muscle wasting through myokine’s and interleukin’s production [164, 165]. However, it should be noticed that in older people, while moderate physical activity mitigates the inflammatory status, resistance exercise is affected by the framework related to the androgen effects on cytokine storm. The variations depend on the specific age, exercise intensity, and individual health status. After a single bout of resistance exercise, older people exhibit a less pronounced increase in cytokine levels, as compared with younger individuals. Anyhow, regular and moderate physical activity is associated with a favorable androgen to cortisol ratio and lower circulating ILs level [165].

Thus, in dissecting the role of the sex hormones, particularly the androgens, it should be noticed that numerous immune cells and, more generally, the immune environment play a role in skeletal muscle wellness. Among the multiple factors fostering the development and progression of sarcopenia during muscle senescence, an unbalanced secretion of pro- or anti-inflammatory cytokines might play a major role (*reviewed in* [166]). This makes more complex the overall impact of sex steroids on sarcopenia, as estrogens and progestins increase, for instance, the immune response, while androgens act in opposite way. Differences between sexes in lymphocyte cytotoxic response, which could contribute to sex-specific differences in the progression of sarcopenia, have been thoroughly described [167]. The interplay between immune response and androgens is also supported by findings concerning their role in cancer immunotherapy efficacy [168]. In example, AR dampens the immunotherapy efficacy in melanoma, likely through its action in natural killer cells [169].

**Fig. 2:**
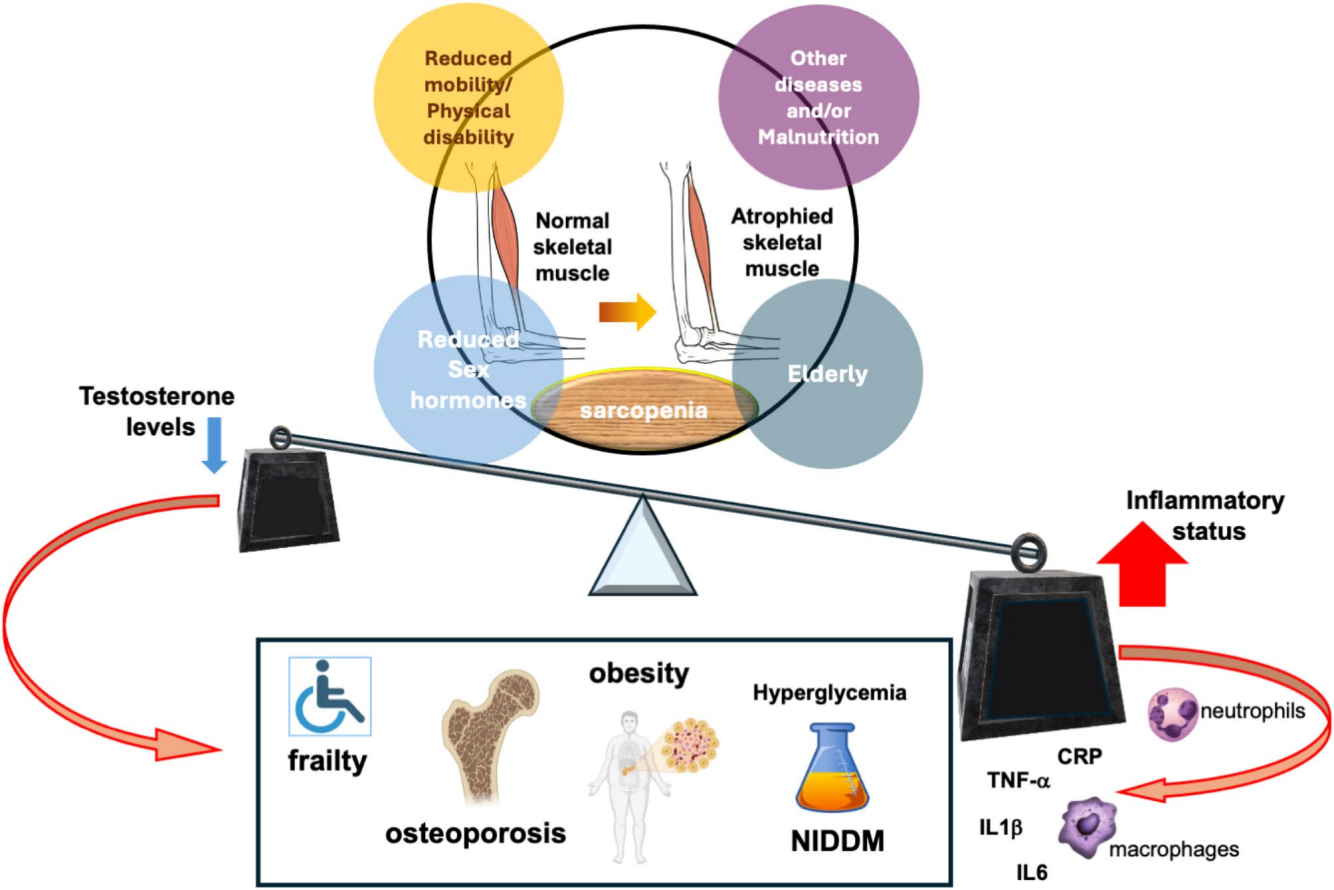
Testosterone, Inflammation, and Sarcopenia: an intricate network. Sarcopenia is often associated with aging, reduced mobility, physical disability, metabolic disorders and lower sex hormone levels. Declining testosterone levels contribute to a pro-inflammatory state, marked by elevated CRP, TNF-α, IL-1β, and IL-6. This inflammatory milieu exacerbates frailty, osteoporosis, obesity, and insulin resistance, thus increasing the risk of fractures and disability

In summary, the findings so far discussed point each other to the role of androgen/AR pathway impairment in skeletal muscle wasting, sarcopenia progression, obesity, insulin-resistance, inflammation and immuno-senescence. A better understanding of this intricate network (Fig. 2) might suggest new approaches for therapeutic interventions, emphasizing the importance of precision hormonal therapies and tailored exercise programs in aging people. In the subsequent section, we will discuss the *pros* and *cons* of the most recent utilized therapies in sarcopenia patients.

### Targeting the androgen/AR axis in sarcopenia: therapeutic potential and challenge

Pharmacological and clinical approaches to sarcopenia remain limited and often controversial. Among potential interventions, the role of androgens is increasingly recognized. Multiple randomized controlled trials have investigated the efficacy as well as the adverse effects of testosterone replacement therapy (TRT) in clinical settings (Table 1).

**Table 1:** Clinical trials using AR agonists or antagonists in patients with impaired muscle function or sarcopenia. DHEA: dehydroepiandrosterone; 11KA4: 11-Ketoandrostenedione; TRT: testosterone replacement therapy; ADT: androgen deprivation therapy; PC: prostate cancer

Intervention/treatment	Study name	Condition/disease	Development stage	Study number
Androgen replacement	Pathogenesis of Sarcopenia and Metabolic Changes in Aging	Sarcopenia	Completed	**NCT00254371**
AndroGel (Testosterone gel), Anastozole (Aromatase Inhibitor)	Effect of Aromatase Inhibition Versus testosterone in older Men with low testosterone: randomized-controlled trial	Hypogonadism, Diabetes, Sarcopenia, Osteoporosis, depression	Completed	**NCT00104572**
DHEA, 11KA4	DIMOXIS	Insulin resistance, Endocrine System Disease	Recruiting	**NCT05263557**
ADT	Investigation bone and skeletal muscle interaction in man with prostate cancer	PC	Completed	**NCT03386812**
Testosterone Undecanoate + exercise training	Does human skeletal muscle possess an epigenetic memory of testosterone?	Healthy Aging, Age-related Sacopenia, Testosterone Deficiency	Phase II/III	**NCT05964920**
Testosterone enanthate	A study to evaluate a skeletal-muscle microbiopsy technique with dynamic proteomic measurement in healthy male volunteers	Cachexia	Completed	**NCT01962454**
Testosterone enanthate/ Finasteride	Testosterone plus Finasteride treatment after spinal cord injury	Spinal cord injury, Trauma, Nervus system, Wound and injures, Gonadal Disorders, Endocrine System Disease, Hypogonadism, Genital disease (Male)	Completed	**NCT02248701**
AndroGel (Testosterone gel)	The Testosterone Trial in Older Men	Andropause	Completed	**NCT00799617**
Testosterone gel	TOM: Testosterone in Older Men with Sarcopenia	Sarcopenia, Hypogonadism, Muscular Diseases	Terminated	**NCT00240981**
AndroGel (Testosterone gel)	A Study to Evaluate the Effect of TRT on the Incidence of Adverse Cardiovascular Events (MAGE) and Efficacy Measures in Hypogonadal Men (TRAVERSE)	Cardiovascular disease, Hypogonadism	Completed	**NCT03518034**
MK-0773	A phase IIA randomized, placebo- controlled clinical trial to study the efficacy and safety of the selective androgen receptor modulator (SARM), MK- 0773 in female partecipants with sarcopenia	Sarcopenia	Completed	**NCT00529659**

In healthy men, supraphysiologic doses of testosterone significantly increase lean body mass, muscle size, and strength, particularly when combined with resistance training. These findings highlight the synergistic effect of androgen therapy and exercise [170] and underscore the potent anabolic properties of testosterone. However, they also raise concerns regarding the long-term safety and ethical implications of its use.

Clinical trials have reported conflicting outcomes regarding TRT in older men. On one hand, TRT has been shown to improve sexual function, mitigate depressive symptoms (NCT00799617) [171], increase bone mineral density [172], and correct anemia [173]. However, the recommended approach is to maintain testosterone levels within physiological plasma concentrations to minimize adverse effects [174]. On the other, in older men exhibiting mobility limitations, TRT has been associated with an increased risk of cardiovascular events, including hypertension, edema, myocardial infarction and stroke, raising concerns about its long-term cardiovascular safety in the aging population (NCT00240981) [175]. A recent clinical trial involving men with hypogonadism and a history of cardiovascular disease, however, reported that the incidence of major adverse cardiac events following TRT was not significantly different from that observed in the placebo group. These data were obtained over a mean follow-up period of 22 months (NCT03518034) [176], leaving the long-term safety profile uncertain.

Despite these risks, short-term androgen administration may have promising applications beyond aging-related hypogonadism. Particularly, evidence suggests that optimal outcomes in sarcopenia management arise from the combination of pharmacological interventions with physical activity [174]. Combination of TRT with training regulates myogenic gene programming, enhances protein turnover and improves muscle function [177]. Such synergies emphasize the importance of lifestyle modifications, including regular exercise and dietary optimization, to amplify the benefits of androgen-based therapies. These findings highlight the need for rigorous patient selection for TRT and the close monitoring of cardiovascular events during treatment. Furthermore, the evidence remains inconsistent, as the potential benefits of TRT are often accompanied by other iatrogenic effects, including erythrocytosis, benign prostatic hyperplasia, gynecomastia, and an increased risk of prostate cancer [178, 179]. Other large-scale, long-term studies are required to fully elucidate the risk-benefit profile of TRT in older people.

Given the controversial effect of androgen-based therapies, SARMs have emerged as a promising alternative. They offer tissue-specific modulation of AR activity and minimize systemic side effects, while providing therapeutic benefits to musculoskeletal tissue [180]. GLPG0492 [181] and SARM-2 F [182] increase, for instance, muscle mass and reduce the amount of adipose tissue, although their long-term efficacy and impact on muscle strength remains uncertain. Unfortunately, many SARMs are so far stalled in phase II clinical trials limiting their large-scale implementation or trials are no longer recruiting patients due to the lack of results. As an example, the SARM MK-0773 (NCT00529659) increases the lean body mass, but its administration was associated with gastrointestinal disorders and an increase in mortality rates [183].

Interestingly, trenbolone, a synthetic androgen widely used by bodybuilders, mimics the SARMs effects and promotes skeletal muscle trophism and bone growth, while reducing adiposity. Notably, it exhibits limited effects in prostate tissue. Because of its resistance to 5α-reductase, trenbolone represents, indeed, a powerful anabolic steroid with a tissue-selective profile [184]. All these features have positioned trenbolone as a lead compound for future therapies, although its broader applicability still requires investigation, because of the lack of long-term safety data [184].

In conclusion, while testosterone- and SARMs-based therapies hold significant promises for sarcopenia treatment, their clinical adoption requires further refinement. Future studies should focus at elucidating the intracellular sites of action, improving safety profiles and establishing long-term efficacy of these compounds. When integrated with tailored exercise regimens and nutritional strategies, these interventions might improve muscle health, enhance the quality of life and mitigate the burden of chronic, non-communicable diseases in aging populations.

## Conclusions

Nowadays, slowing down aging is a huge challenge that strongly influences the scientific community, from basic biologists to clinicians. Concerning the skeletal muscle, progression towards aging and acquisition of a sarcopenic state leaves still pending many issues that need to be further exploited. Aging favors, indeed, the onset and progression of chronic diseases and the establishment of a low-grade inflammation that impinges on inflammatory mediator’s release and availability of growth factors as well steroid hormones. An in depth understanding of this intricate picture might open new paths to restore a more youthful local microenvironment. Given the importance of androgens and their role in skeletal muscle, a better understanding of their connection with other soluble and non-soluble signals that influence muscle wellness might bring out combinatorial therapies based on the use of molecules with anti-inflammatory properties (including natural compounds) and AR agonists devoid of side effects in sarcopenia treatment. In this regard, it should be noticed that in silico screening methods recently identified a small molecule, AP503, which activates the membrane androgen receptor GPR133 and shows beneficial effects on muscle strength, leaving unaffected the other actions mediated by AR in mouse model. GPR133 might represent a clinically actionable target in sarcopenia’s treatment [113]. Similar approaches, together with physical and lifestyle interventions, might maintain muscle’s strength and activity, while offsetting the economic burdens of older people.

## Data Availability

No datasets were generated or analysed during the current study.

## Declarations

### Declarations of Generative AI and AI-assisted technologies in the writing process

Authors did not use generative artificial intelligence (AI) and AI-assisted technologies in the writing process.

## Abbreviations

ICD-10: International Classification of Diseases
AR: Androgen receptor
ER: Estrogen receptor
PR: Progesterone receptor
GH: Growth hormone
SARMs: Selective androgen receptor modulators
DHT: Dihydrotestosterone
ARE: Androgen-responsive elements
ChIP: Chromatin immunoprecipitation
ChIP-qPCR: ChIP-quantitative polymerase chain reaction
Mef2: Myocyte enhancer factor 2
Mef2c: Myocyte enhancer factor 2 C
ARKO: AR knockout
MCK: Muscle creatine-kinase
HSA: Human α-skeletal actin
MyoD: Myoblast determination protein 1
cARKO: Myofiber-specific ARKO
Mylk4: Myosin light chain kinase 4
fmARKO: Fast-twitch muscle-specific ARKO
ROS: Reactive oxygen species
Fbp2: Fructose-bisphosphatase 2
Pcx: Pyruvate carboxylase
Pfkfb3: 6-Phosphofructo-2-kinase/fructose-2,6-biphosphatase 3
GSN: Gelsolin
ADF: Actin-depolymerizing factor
Hic-5: Hydrogen peroxide-inducible clone-5
ARA55: AR co-activator Hic-5
PXN: Paxillin
GR: Glucocorticoid receptor
SRC: Steroid receptor coactivator
NCOA: Nuclear receptor coactivators
PGC-1: Peroxisome proliferator-activated receptor gamma coactivator-1
PRC: PGC-1-related coactivator
HDACs: Histone deacetylases
NCoR: Nuclear Receptor Co-Repressor
PKA: Protein kinase A
PKC: Protein kinase C
PLC: Phospholipase C
Src: Sarcoma kinase
PI3Ks: Phosphoinositide 3-kinases
MAPKs: Mitogen-activated protein kinases
IP_3_: Inositol trisphosphate
PTX: Pertussis toxin
MEK: Mitogen-activated extracellular signal-regulated kinase
ERK: Extracellular signal-regulated kinase
mTOR: Mammalian target of rapamycin
FOXO3a: Forkhead box protein O3
mTORC1: mTOR Complex 1
FAK: Focal adhesion kinase
IRSs: Insulin receptor substrates
AMPK: AMP-activated protein kinase
IGF-1: Insulin-like growth factor 1
IGF-1R: Insulin-like growth factor 1 receptor
4E-BP1: Eukaryotic translation initiation factor 4E-binding protein 1
eIF4E: Eukaryotic translation initiation factor 4E
S6K1: Ribosomal protein S6 Kinase beta 1
MAFbx: Atrogin-1/muscle atrophy F-box
MuRF1: Muscle RING finger 1
mAR: Membrane AR
aGPCR: Adhesion G-protein coupled receptor
GPR 133: G protein-coupled receptor 133
Fln: Filamin
Rac 1: Ras-related C3 botulinum toxin substrate 1
Drp1: Dynamin-related protein 1
TGF-β: Transforming growth factor-β
SMAD: Small mothers against decapentaplegic
SMAD4: SMAD family member 4
Pax3: Paired Box 3
ADT: Androgen deprivation therapy
IL-6: Interleukin-6
LPL: Lipoprotein lipase
TNFα: Tumor necrosis factor-α
IL-1β: Interleukin-1β
IL-10: Interleukin-10
CRP: C-reactive protein
TRT: Testosterone replacement therapy
