# Correction: Androgens as the “old age stick” in skeletal muscle

**DOI:** 10.1186/s12964-025-02234-8

**Published:** 2025-05-15

**Authors:** Giulia Gentile, Ferdinando De Stefano, Carmela Sorrentino, Rosa D’Angiolo, Carmine Lauretta, Pia Giovannelli, Antimo Migliaccio, Gabriella Castoria, Marzia Di Donato

**Affiliations:** Department of Precision Medicine, University of Campania “Luigi Vanvitelli”, Via L. De Crecchio 7, Naples, 80138 Italy

**Correction: Cell Commun Signal 23**,** 167 (2025)**

10.1186/s12964-025-02163-6

Following publication of the original article [1], the authors identified an error in the author group. The given names and family names were erroneously transposed.

The author group has been updated above and the original article [1] has been corrected.
